# Negotiating support from relationships and resources: a longitudinal study examining the role of personal support networks in the management of severe and enduring mental health problems

**DOI:** 10.1186/s12888-020-2458-z

**Published:** 2020-02-07

**Authors:** Helen Louise Brooks, Penny Bee, Karina Lovell, Anne Rogers

**Affiliations:** 1 Department of Health Services Research, Institute of Population Health Sciences, University of Liverpool, Liverpool, UK; 2 Mental Health Research Group, Division of Nursing, Midwifery and Social Work, Faculty of Biology, Medicine and Health, School of Health Sciences, Manchester Academic Health Science Centre, University of Manchester, Manchester, UK; 3 Greater Manchester Mental Health NHS Foundation Trust, Manchester, UK; 4 NIHR CLAHRC Wessex, Faculty of Environmental and Life Sciences, University of Southampton, Southampton, UK

**Keywords:** Mental health self-management, Personal support networks, Personal communities, Qualitative, Valued activities, Relational work

## Abstract

**Background:**

Personal communities or personal support networks comprise a variety of social ties considered important to individuals in their everyday lives. This set of active and significant ties influence the capacity to manage mental health problems because of the potential to access social support. However, little is known in the context of people’s everyday management of mental health about how relationships with people, places, objects and activities are navigated and negotiated. This study aimed to explore the nature and negotiation of support from personal communities in the everyday management of severe and enduring mental health problems.

**Methods:**

A longitudinal qualitative study undertaken in the UK incorporating 79 interviews with 29 participants based on personal network mapping. 29 users of mental health services with a diagnosis of severe and enduring mental illness were interviewed at three time points. Data was analysed using an inductive thematic approach underpinned by the Network Episode Model.

**Results:**

The presence and maintenance of interpersonal trust was a fundamental condition of the relational work required to develop, undertake and sustain relationships with others. Whilst relationships with spouses, family members and friends were generally viewed positively, the work required to engage human others was contingent, vicarious and overlain with felt and enacted stigma. Developing relationships with others was hindered by a lack of confidence fuelled by the experience of mental illness and a fear of rejection or failure. By contrast, weaker ties and inanimate objects and places offered and provided a sense of reliability and security. Strategies employed by participants in order to garner sufficient support for condition management in the light of these particular challenges are illuminated by the discussion of who and what is relevant and valued in personal support networks.

**Conclusions:**

Access to valued activities, hobbies and things should be considered alongside human relationships in providing a means of ongoing support and resource for the everyday management of life for those experiencing severe and enduring mental health problems.

## Background

Traditional approaches to mental health management have largely ignored the possibilities that emanate from accessing resources and connecting to support within personal support networks which are likely to be relevant to the daily experience of mental health care and self-management [1]. The recognised need to move towards user-centered mental health care is difficult to realise in routine practice settings due in part to a lack of incorporation of the expressed needs of service users, which fails to align service provision with the everyday reality of managing a long term mental health problem [1, 2].

One way of engaging with the latter is through the analysis of personal support networks - a variety of social ties considered important to individuals which enable the mobilization of resources to support people in their everyday lives – which are thought to influence recovery from, and potentially the management of, mental health [3]. Recent systematic reviews demonstrate the positive impact of social network interventions on health outcomes for people with a diagnosis of a severe mental illness [4, 5] Adequate, diverse social networks can mediate the effects of social isolation and loneliness, enhance self-management, circumvent the need for formal health services [6] and hospitalization [7] and reduce suicidality [8].

The Network Episode Model (NEM) provides a theoretical underpinning to the consideration of social networks to support people living with severe and enduring mental illnesses such as psychosis, schizophrenia, bipolar disorder, or personality disorder [9–11]. Moving away from individualistic approaches, this theory asserts that the myriad of activities that people do in conjunction with their social networks to manage a mental health condition are dynamic social processes responsive to changes in external circumstances [12]. NEM and other social network approaches explicate the mobilization of social networks (both lay and professional) in response to health problems which extend beyond an individual’s own capacity to self-manage [13, 14]. Medical sociologists have highlighted the bi-directional role of culture and habitus (ingrained habits, skills and dispositions [15, 16]) in relation to the activation of social networks and enactment of health behaviours [17]. An individual’s propensity to mobilise support from others is likely therefore to operate within the confines of extant cultures, habitus and resources [18]. Social network activation can have both positive and negative outcomes and, as such, is thought to play a significant role in self-management and recovery [9, 10].

People diagnosed with severe mental health problems have previously been found to possess social networks of comparatively smaller size and of poorer quality, reducing people’s capacity to access social capital and support [19, 20]. Longitudinal research has demonstrated that social networks can be large, diverse and supportive during periods of initial crisis but that levels and quality of contribution by networks diminish over time [21–23]. Such deficits have been attributed to symptomology including reduced motivation and capacity for interpersonal interactions [24], labelling [25] and both felt and enacted stigma [26, 27]. Additionally, the perceived burden associated with caring for people with serious mental health conditions may further contribute to reductions in social network support [28].

Networks of diminished quantity and quality have the potential to exacerbate existing social disadvantage reducing the opportunities and capacity to develop new or sustain existing social connections which might otherwise be used to manage mental health symptoms [5]. However, recent evidence suggests that self-imposed isolation or network restriction particularly during times of crisis can be viewed as an active strategy for managing mental health through the provision of time and space for healing and as a way of regaining strength [29]. Similarly, longitudinal studies demonstrate that people experiencing the first onset of serious mental illness actively reconstruct the ways in which they engage with social networks (reduced contact or termination of social contacts) to avoid having to tell people about their illness, to reduce interpersonal conflict and lessen burden to themselves and others [30].

Through the identification of reciprocal relationships and the sustaining features of supportive networks [3] personal network mapping provides an heuristic device for ascertaining a broader understanding of individuals’ lives and the management of mental health in everyday settings. Network diversity is important in relation to the capacity to leverage resources [31]. In relation to mental health, ‘formal and sparse’ networks – those with fewer social ties, higher proportions of professional network members and lower engagement in social activities - have been associated with lower subjective wellbeing compared to other types of more socially diverse networks [32]. Less is known about what is involved in retaining and utilising networks as part of on-going management and the work entailed in mobilising and using personal support networks in everyday settings over time.

Social networks create both opportunities and constraints for people in terms of accessing and using resources to manage mental health [33] . The association of increased network resources with better mental health implicates viewing the configuration of connections to people, places, locations and meaningful activities as the building blocks for personal mental health recovery [18, 32]. Currently, relatively little is known about the *value* that people attach to network members, *how* decisions are made about connecting to and sustaining different types of network relationships or the *work* required to navigate and negotiate such relationships. The interactional strategies that people employ to develop and sustain relationships with social network members constitute ‘relational’ work [34]. This includes the ongoing effort people deploy in differentiating, establishing, maintaining and changing interpersonal relationships [35]. Specifically, relational work involves identifying and linking to relevant networks members, the ongoing negotiation of relationships within networks (e.g. agreement over roles and relationships as well as preferred modes of interaction) and the development of a collective ability to effectively undertake desired behaviours based on shared understanding of requirements and efforts [35].

In this paper we use a personal support network approach to explore the nature and negotiation of support in the everyday management of severe and enduring mental health problems. We use the notion of ‘personal support network’ or ‘personal community’ to refer to a group of network members who contribute to an individual’s well-being and mental health management through providing support, approval validation and a sense of value [33].

Drawing on the Network Episode Model and using longitudinal personal network mapping combined with in-depth qualitative interviews, this study aimed to explore how people with a diagnosis of severe mental illness obtain and negotiate support from personal networks. The NEM explicates that the management of health conditions is a dynamic social process responsive to changes in external circumstances. We therefore collected longitudinal data in order to explore the contributions of personal support networks to mental health management over a 12-month period and to compare relationships with different network members over time.

## Methods

This study was part of a larger programme of research to enhance service user and carer involvement in mental health services through the development and evaluation of a training programme for mental health professionals [36]. People were eligible to take part in the trial if they were aged over 18 and had a diagnosis of a severe and enduring mental health problem (including psychosis, bipolar disorder, schizophrenia and personality disorder). People were excluded if they did not have capacity to provide informed consent or were considered by care teams to be too unwell to participate. A short film detailing the parent trial and its findings can be found here: http://research.bmh.manchester.ac.uk/equip/mainfindings

A longitudinal, qualitative study was undertaken incorporating 79 semi-structured interviews. Interviews centred on personal network mapping with 29 participants and were carried out at three time points (0, 6 and 12 months).

### Context and sampling

Participants were eligible to take part in the study if they were a mental health service user from a community secondary mental healthcare service taking part in an ongoing randomised controlled trial [36].

Eligible participants (purposively sampled in relation to gender and geographical area) were sent written invitations, which included a participant information sheet and consent to contact form. Interested parties completed the consent to contact form and returned this to the study team who contacted potential participants to discuss involvement further and arrange a convenient time and date for baseline interview.

Saturation was a standing item on the agenda for data analysis meetings. Data collection stopped when consensus was reached amongst the research team that data saturation had occurred. This was agreed initially after 25 interviews had been conducted and a further 4 were undertaken to ensure that further data collection was not necessary.

Forty-seven service users expressed an initial interest in taking part. 29 mental health service users with a clinical diagnosis of severe and enduring mental illness (psychosis, bipolar disorder, schizophrenia and personality disorder) from seven Mental Health Trusts in the UK consented to take part in the study. Reasons for non-participation included non-response and participants no longer wishing to take part because of illness or changes to personal circumstances. Further detail on study participants can be found in Table 1.

**Table 1:** Participant information

Gender	Male	13
	Female	16
Has a spouse/partner?	Yes	13
	No	16
Ethnicity	White	27
	Non-white	2
Network size	Average	9
	Range	3–16
Types of network members:		Total (average per network)
	Spouses	13
	Family members	65 (2.3)
	Friends	45 (1.6)
	Professionals	46 (1.6)
	Other	85 (3)
Mental Health Trust	Trust 1: North West of England	12
	Trust 2: East Midlands	5
	Trust 3: Northern England	3
	Trust 4: Midlands	7
	Trust 5: North West of England	2
Trial allocation	Control arm	11
	Intervention arm	18
Completed interviews	Baseline	29
	First follow up	26 (90%)
	Second follow up	24 (83%)

### Data collection

**Fig. 1:**
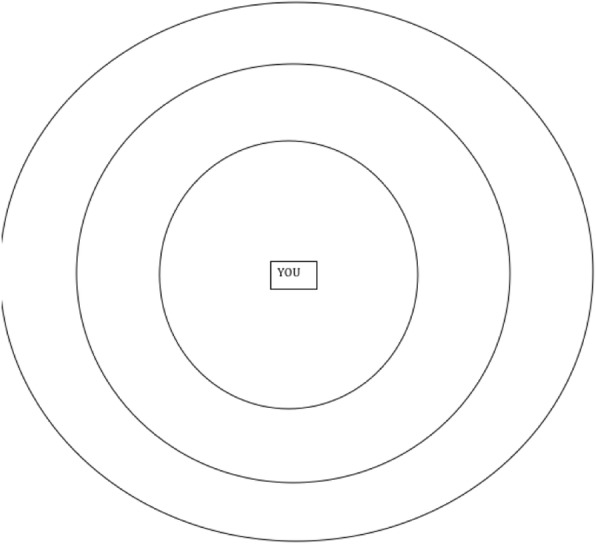
Example network diagram

Data were collected using in-depth semi-structured interviews at three time points (0, 6 and 12 months) undertaken by the lead author. Interviews started with personal network mapping of important people, places, and activities identified as relevant to self-management using a network diagram (Fig. 1). Participants were asked to place identified network members in one of three concentric circles based on importance. Network diagrams were filled in by the interviewer in collaboration with the participant. The interview then qualitatively explored the *value* that people attach to each network member, *how* decisions were made about connecting to and sustaining different types of network relationships and the *work* required to navigate and negotiate such relationships [33].

Interviews were carried out between August 2014 and April 2017 in participants’ homes or over the phone depending on individual preference and lasting an average of approximately 60 min (range: 15–70 min). Four participants expressed a preference for telephone interviews and consent forms for these participants were returned by the post in advance of interviews.

Participants were not restricted in terms of the type of network member or the size of personal support network with the onus placed on participants to identify sources of support that were important to them. Identified network members included friends, family members, health professionals, pets, hobbies, places, activities and objects. This was supplemented by additional questions drawn from the literature which were designed to explore the role and function of different network members in relation to the managing of mental health in everyday settings [29, 32, 35, 37–39]. See also the Appendix. During follow-up interviews, network maps were revisited and any changes in network size or function and the reasons for these were explored.

### Data analysis

Interviews were recorded using an encrypted digital recorder and transcribed verbatim by an experienced transcription company before being anonymised and allocated to a member of the research team for analysis. NVIVO V.11 was used to analyse the transcripts using inductive thematic analysis [40]. Data also included network maps completed by participants and revisited over the course of follow-up interviews. Transcripts were firstly read and re-read alongside the network diagrams by two authors (HB and AR) to ensure familiarisation with and immersion in the data. Both authors independently identified inductive codes in the data from interviews with the first ten participants. They then met to discuss the coding process, to identify any discrepancy in coding, remove duplicate codes, merge similar codes and to organise codes into overarching themes which formed the basis of the emerging analytical framework. HB then applied the framework to the remaining transcripts supported by NVIVO which resulted in further minor modifications. The analysis was then presented to the wider study team to ensure interpretations were grounded in the data and to allow for final refinements to be made. Agreement was then reached that the final framework was considered representative of the entire data set.

HB is a Senior Lecturer in Health Services Research, PB a Professor of Health Services Research, KL a Professor in Mental Health and AR a Professor of Medical Sociology. No authors had any prior therapeutic relationships with participants nor were they known to any of the research team. The starting point for the research was one informed by the value of personal communities for the management of chronic health conditions. All interviews were conducted by HB a senior lecturer and post-doctoral researcher with significant qualitative experience.

## Results

Qualitative analysis interpreted four overarching themes from the data: familial and spousal support: the salience of guilt and burden in kin relationships, the friendship paradox: negotiating the precariousness of non-kin ties*,* the limited and rationed role of mental health professionals and the significance of activites, objects, places and groups in identified support networks. Each theme will be presented in turn supported by thick descriptions of interpreted themes and direct quotations from the data to support interpretations.

A fundamental condition of the relational work required to develop, undertake and sustain relationships centred on the development of interpersonal trust. Self-censorship through fear of stigma meant people could be reticent and cautious in how they presented themselves and what they were willing to reveal to others which extended to all types of network members.

Consistency of and diversity within personal support networks over the 12-month period were considered important to participants in relation to managing mental illness and maintaining identity outside of being someone with a mental health problem. The consistency and subsequent reliability of relationships varied between different types of network members which is discussed in presented themes.

### Familial and spousal support: the salience of guilt and burden in kin relationships

#### Relational work within intimate relationships

Just under half of participants (13/29) reported having a spouse or partner who appeared to play a fundamental role in supporting people with their mental illness. Identified support was consistent over the 12-month data collection period and was often taken for granted as an implicit part of these relationships without the need for requests for support to be explicated.

***What does your partner do for you?***

*Everything from just the normal day-to-day stuff you do as a couple, do you know what I mean, like the happy times and all that lot, she’s probably one of the only people that knows pretty much everything that’s happened what I struggle with day to day; and she knows me better than I do sometimes when – do you know what I mean – when I’m struggling or when I’m not myself, or that sort of stuff, she’s, like, the first person to kind of notice it and kind of say, 'what can I do to help?'.*

*I don’t think I would be alive if I didn’t have her really, which is quite a big statement to say; but when things were really tough my family, all my nieces and nephews didn’t stop me from attempting to take my own life, it was her that kind of was that person to kind of cling on to and stop me.***ID28, female, 1st time point**

Some of those with spouses or partners reported that despite these positive contributions, the experience of mental health problems could concomitantly cause frustration for partners or place strain on relationships especially if spouse/partners were considered to lack understanding about mental illness. This pressure appeared to be a result of the level of support people required from their spouse/partner which was considered burdensome and altered dynamics within relationships placing the spouse in a more ‘caring’ role. This had negative connotations such as guilt and shame and reduced the likelihood that people would ask for additional support from these network members.

*My partner, he’s the one person that I … I speak to most of the time, too much really, sometimes because it’s a bit of a burden for him, but he, sort of, encourages me, he tries to, sort of, just bring me back and tell me to, sort of, you know, take things one step at a time, he encourages me, he, erm, tries to bring me back really from, sort of, because I get … my self-esteem is quite low***ID24, female, 1**^**st**^**time point**

*Did he help you get through those difficult … that difficult period?*

*I don’t think he did, to be honest, he’s bloody useless.*

*He’s useless [laughs], okay, in what ways?*

*He just gets frustrated and angry about things, he cannot understand why I feel so low. When I’ve got so many things going on in my life. he just can’t get to grips with it and he gets frustrated and he gets frustrated at the system as well.***ID27, female, 2**^**nd**^**time point.**

Participants described how living with mental health conditions made establishing and sustaining intimate relationships difficult or in some cases impossible. For example, one participant cited the side effects of his medication (e.g. erectile dysfunction) as a direct barrier to establishing relationships with a partner - ‘it’s very difficult with erectile dysfunction to actually start a relationship’ **ID20, male, 1st time point**. Others reported difficulties in knowing when to divulge mental health status if they met new partners during the 12-month period out of a fear of losing these relationships.

*I haven’t divulged my mental health background, because I think that that’s really difficult for people to handle; and I fear that he’d probably think, oh no, she’s not right, she’s a bit of a nutter – in inverted commas – so I haven’t divulged that. And I don’t know when the right time would be to do that; I don’t know, I don’t know about that. And I worry that that would have an impact, a negative impact, on my relationship***ID15, female, 2**^**nd**^**time point**

#### Relating to family members

Whilst generally viewed positively and as reliable sources of support over the 12-month data collection period, the negotiation of support from family members could be complicated. It appeared some family members were sometimes being placed in personal communities because of attributions of love rather than the actual support received. Participants were often wary about asking for help from family members because of concerns around overburdening then either practically or emotionally. Participants acknowledged that family members had their own lives and their own responsibilities to deal with and did not want to create additional burden for them. Additionally, family members could overstep interpersonal boundaries in relation to managing mental health conditions which had negative connotations.

*Because I suppose my mum, I wouldn’t want to bother her and we’re not very close really. I never really have been. She loves me and everything but I don’t know. She’d ask too many questions or … And my sister, again, we’re not that close. We are but I wouldn’t go to her. And my dad, well I love my dad to bits but he’s very opinionated. And he can annoy me sometimes.***ID29, female, 1**^**st**^**time point**

Often family members were reported to support participants out of a sense of familial responsibility with little understanding of mental illness which detracted from the value attributed to this form of support.

*Well, my sister, my older sister she lives in Norfolk … she helps me very much financially. She can’t spend a lot of time here so I … I think she feels that … she’ll … she’ll often buy me underwear and socks, tee-shirts. Like I say, she does really try and help but she doesn’t … she doesn’t quite understand, I don’t think about how debilitating having a mental illness can be sometimes.***ID22, male, 1**^**st**^**time point**

Kin relations were viewed as supportive where there was shared understanding of a person’s experience of mental illness. Familial relationships built on understanding and honesty could provide validation of past experiences and an important alternative point of view with which to challenge negative thoughts or feelings.

*He sort of understands, he understands my past, he confirmed some of it, which makes me feel good. Not good as in, you know, but relieved that it's not just me, I'm not imagining it, which is positive, and he's completely honest with me. He'll talk things through with me. He will get me to look at things from another point of view just to make sure, be devil's advocate a little bit, so from that point. And I know he loves me.***ID24, female, 2**^**nd**^**time point.**

Participants in the current study; however, often identified difficult and sometimes fractious relationships with family members. An inability to be open and honest with family members about mental health detracted from the interpersonal trust considered fundamental for the leveraging of support from network members.

*And it’s recently that, problems have been identified between mine and Mum’s relationship, but I suppose putting a strain on that without her knowing, because she doesn’t know about the childhood abuse so it’s a bit like … it’s only recently that I’ve processed that to then question that relationship between me and Mum***ID28, female, 1st time point**

*When you’re like this you lose a lot of trust in people, family as well, you know, because you tend not to tell them things and the less you tell them the less they can say. It’s like my daughter, you know, I’ve tried talking to her and she … all she … I mean, she’s, sort of, semi alcoholic and she just keeps saying, oh, get a grip, get a grip and you just think, mm, I’ve tried getting a grip and I’ve just got to the stage where I just want somebody to talk to and listen to me.***ID23, male, 1**^**st**^**time point**

*I say really unpleasant, err, nasty things to people and then when my mood starts to level out I sometimes start to remember how I've treated people, and so that, erm, impacts on my wellness where I start to feel incredibly guilty … I couldn't speak up, erm, so I started to feel dumb to or over spoken to by my family because I thought well, I can't really voice my opinion because I upset them before, erm. So, I became very, err, low in terms of my self-esteem and my confidence, and that was primarily because of how I'd behaved when I was unwell and how, when I started to recall how I'd behaved.***ID15, female, second time point.**

Others felt that family members asked for too much support from them which they felt obliged to give out of familial responsibility but which exacerbated mental illness (e.g. provision of emotional support to others or caring for children/grandchildren). Despite these identified difficulties, relationships with family members remained fairly stable over the 12-month period.

*So, she’ll [sister] let off steam and she says, well, if I can’t let off steam with you, who is meant to be my nearest and dearest, who … who, you know, how can I, sort of, unburden myself a bit. So, I sometimes find it quite difficult.***ID24, female, 1**^**st**^**interview**

*I think not having my grandson for a little while helped, because I was having him quite a lot due to his mum’s work commitments, and I just said I can’t cope with him, and I couldn’t cope with him all the time like I was having him. And it tailed off to the fact I wasn’t having him and I seemed to pick up then.***ID27, female second time point**

Conversely, relationships with younger network members such as nieces and nephews were often considered free from these complexities where they were not bound up in caring responsibilities and were an important distraction from everyday life.

*I suppose with my mum now, she’s constantly on edge, if I say I’m not feeling great today, she’s hyper vigilant and she’s thinking that things are going to deteriorate; whereas the kids, they’re not bothered, are they, they don’t know … Yeah. So, yes, but it kind of, yeah, gives you a different focus, so you don’t have to think about, if you are feeling a bit rubbish then it’s all right because you’re doing stuff for the kids and it’s passing the time.***ID28, female, 1st time point**

### The friendship paradox: negotiating the precariousness of non-kin ties

Friendships provided valued support and connection to the social world where these relationships were functional and not overbearing. Friendships; however, were more flexible and subject to change over the 12-month period when compared to spousal or familial relationships.

*That’s valuable in itself, it’s not a pushy overbearing type of friendship. I have known her for twenty-five years, there are times when we have not spoken for, I mean, we have had periods where we have not spoken for three years, but we can pick up where we have left off. So, there is no, what’s the word, I don’t know, I can’t think of the word at the moment, but there is no pressure in that relationship. There is no pressure to think well, oh, you know, I have got to ring you or I have got to speak in this particular way or I have got to feel guilty about not contacting her.***ID15, female, third time point**

These types of relationships were considered particularly beneficial if friends shared similar conditions and/or experiences (e.g. shared childhoods), which contributed to a shared sense of understanding. Friendships did not need to be traditional face-to-face friendships and often it was the more distal friendships (e.g. online friends) or weaker ties (online acquaintances or members of the wider community such as shop owners or taxi drivers) that were viewed most positively because of reduced expectations and responsibilities for reciprocity.

*Drop in friends, I see every week, so it’s important to me, even though they’re not all close to me, you know, we’re … we’re all in the same boat really, so it helps.*

**ID11, male, time point 1**

*So, sometimes I just want to go out and have a laugh, you know, go out and have a drink or whatever and just...that’s it. We talk about nothing but football and whatever, you know.***ID12, male, 2nd time point**

However, friendships were often considered to be unsustainable for participants because of the relational work required to manage and maintain these. Longitudinal data demonstrated that friends were often the first type of network member to be lost from a network following the period after an acute mental health exacerbation either because they had actively cut contact with them or because they had drifted apart because of a failure of both parties to undertake the work required to sustain relationships. Whilst some participants reported this loss to be negative, most felt that this was necessary to allow them to manage their own condition effectively. Maintaining successful friendships necessitated participants being available to their friends, socialising with them and being willing to provide support to friends should they require it. This was often considered too burdensome for participants particularly when they were feeling unwell and unlike familial relationships there was less obligation to provide this.

*That [friendship] fizzled out as well, I don’t seem to be keeping many friends these days. It fizzled out, it was … I don’t know, with her as well, she was asking too much of me, asking for some of my craft things and I was giving them to her all the time, and she wasn’t giving them back.***ID1, female, 2**^**nd**^**time point**

A small number of participants reported friends had cut contact with them following acute periods of mental illness.

*Friends that I would have spoken to every day I’ve not heard from for two and a half years, and that’s just the fear of not knowing what to say or I’m not sure, really. It’s the stigma and everything.***ID22, male, third time point**

One strategy employed to exploit and mobilise resources within friendships was to have a mixture of different types of friendships in which each contributed a unique source of support.

*I think having good friends around you, you know, and people with various degrees of, what’s the word, I mean sometimes you need friends you can be candid with and sometimes you spend just an hour with, you know. And a good mixture, a good balance of people around me has been a help.***ID28, male, 2**^**nd**^**time point**

Others decided against expending efforts negotiating trust and sustaining relationships with those that they considered to be non-essential relationships and chose instead to focus on garnering support from one or two key network members considered instrumental to managing mental health.

*Is there anyone else who helps you out?*

*No, it is just Laura [health professional] and my husband who I interact with. I don’t need any friends to help me, just my husband. He does most of the things, he cleans the house, takes care of Frankie [child].***ID3, female, 2**^**nd**^**time point**

### The limited and rationed role of mental health professionals within personal communities

Data demonstrated the positive and negative consequences that participants derived from relationships with health professionals. Such relationships could be free from the complexities identified in relationships with friends and family. For example, they would not be subject to the often-complicated family histories people identified in kin relationships, professionals would not burden service users with their own problems and service users would not have to worry about upsetting professionals in relation to disclosures about their mental health.

*Alan [husband] will see a certain amount but I mean, he doesn’t realise what goes on in my head completely. I mean, he … he has to put up with things like how big am I, and, sort of, I can’t do this and things like that. But, sort of, when I get suicidal I can’t necessarily tell him because it upsets him … But it would be [care co-ordinator] and [Psychiatrist] that I'd tell.*** ID1, female, time point 1.**

Participants felt that health professionals should have a de facto understanding of mental health problems because of their professional training, which would promote non-judgemental support which was sometimes seen as lacking in relationships with other people in their networks. Participants described withdrawing from social interaction when acutely unwell highlighting a potential role for professionals in terms of challenging self-imposed insularity.

*I don’t want to explain myself to them [friends] because I don’t think they understand my illness, I don’t think they understand mental illness; so, it’s as if they’re on another planet to me.***ID 11, male, time point 1**

*And you feel sometimes you just need that person that’s there that’s not linked to family or friends, that they’re going to outwardly care as much as what your family and friends would and … [but you won’t] cause them any hassle.***ID12, male, 2nd time point**

*The illness that I have, it … I … it sends you like a hermit. I won’t go out. I won’t do nothing.***ID19, male, 1**^**st**^**time point**

Despite the potential benefits and value attributed to professionals by participants within network diagrams, overall the data identified the limited contribution professionals actually made to the work of managing a mental health condition in everyday settings which remained consistent over the 12-month follow-up period. For most participants, health professionals’ roles rarely extended beyond medication prescription and health surveillance. It appeared the placement of health professionals within network diagrams was often based on a consideration of anticipated or ‘hoped for’ support rather than actual support.

*They ask about the medication, you know, they’d be more interested [if] I’d got a bad elbow...but they don’t seem to mention anything with depth to do with that [mental health], you know …*

*… It was a case of take your medication, watch TV and don’t, err, let anybody jump on you and don’t jump on anybody else.***ID12, male, 1**^**st**^**time point**

*Well Andrew’s, well Andrew’s [care co-ordinator] terrible really because he’ll come and see me at eight o’clock in the morning and then sometimes he can’t come to see me and then sometimes he’s poorly or something happens and he can’t come and see me. And, no he’s very, very nice. I get on with him very well but sometimes he hasn’t got enough time. Because he’s looking after so many people he hasn’t got enough time for everything.****ID26, female, 1***^***st***^***time point***

The limited contributions made by professionals over the 12-month period appeared to be due to insufficient communication and the efforts involved in investing in the relational work required to develop adequate interactions and affiliation. Pre-requisites of the latter included the need for regular access to health professionals who had the time and motivation to invest efforts to get to know service users and develop an understanding of their condition and adequate interpersonal trust. Support workers were considered the most likely to have the time and inclination to undertake this type of relational work and were compared directly as a group of health professionals to psychiatrists and community psychiatric nurses in this regard.

*They [support workers] basically, they know me very, very well. And they can pick up [my mental health] … you know, before it gets worse … … so I find that because they know me well, that I can sort of basically open up to them, you know. They come about twice a week and they just sort of sit with me and talk and then they sort of help me with my housework, you know …*

*… I can trust my support workers. My CPN, she doesn’t know me.***ID2, male, 2**^**nd**^**time point**

The inability to develop desired relationships with professionals was often attributed to a lack of resources within mental health services that meant reliability and consistency of contact was undermined. This was seen as something that had gradually got worse over recent years and that directly impacted on the trust they had in professional relationships.

*And, err, like she’s, she’s a very, very good support worker and she, she does so much and she goes out of her way to help you. But sometimes like, she can make an appointment with me and then she’ll have to cancel because someone that’s poorlier … and I, I fully understand that.***ID4, female, 2**^**nd**^**time point**

Participants reported active attempts from health services to reduce contact between professionals and service users over the 12-month follow-up period through reduced access and an increased focus on discharging people from services. This caused participants concern about future mental health management especially if these roles could not be substituted from elsewhere within personal communities.

*And what about [your contact with] the psychiatrist?*

*She’s backing off a little bit because she’s had to... she [said] something about the powers that be said she can't see patients as frequently. She’s got to see less patients so she comes to see me every six weeks... ...instead of every four. And it’s basically because of the cutbacks not because my needs are changing.***ID1, female, 2nd time point**

In addition to the rationing of support from health services, participants also described rationing their own access of health services because of their concerns about staff being overworked and a perceived vulnerability of health services due to cut backs. High staff turnover meant service users themselves were also less inclined to undertake the often-substantial relational work required because of concerns that this would be wasted if their health professional changed regularly.

*The guy who is my, like, community worker, the support guy that comes to see me, he’s leaving. So, I’m going to get a new one. And then it’s, like, explain all that I go through and all that again. I’m dreading it.***ID19, male, 1**^**st**^**time point**

A further barrier to relationship development with health professionals was the exposure to surveillance, scrutiny and detainment out with an individual’s’ control which threatened to undermine the interpersonal trust required to develop and sustain relationships.

*It took me a while to trust Sarah because she’s a professional and I’m a bit like, all professionals do the same. I’ve had some really bad experience with professionals and safeguarding and all that sort of stuff; and I’ve still even now, when I go to the group I’m still saying to them, I’ve not built that trust back up and it’s going to take me a while to get to that point where I feel like I can trust you again.***ID28, female, 1st time point**

*Professionals can be quite … what's the word, you have to take their opinions on board and that's quite difficult. It seems like it's better if you can take their opinions like you would with a friend or family member, think about it yourself and kind of make those decisions for yourself, with [having] to take their advice on board.***ID24, female, third time point.**

Moral positioning was evident in the presentation of self before mental health professionals which further complicated relationships.

*Yeah, I mean I'm used to it. I'm used to seeing doctors, psychiatrists and having to bare your soul in front of them [laughs].***ID20, male, 1**^**st**^**time point**

### The significance of activites, objects and groups in identified support networks

Identified networks were fairly limited in size (average size 9, ranging from 3 to 16) and participants described an enhanced salience of and value attributed to activities, objects, places and groups. This seemed to be related to a perception of increased ontological security - a sense of order and continuity derived from a person’s capacity to give meaning to their lives and to maintain a positive view of the self, world and future [41] - with these network members. The impact of stigma and the struggle to be in control of emotions, feelings, and behaviour identified in relationships with other humans made these relationships harder to negotiate and thus more likely to be avoided. The circumstances of on-going management also involve having to account for oneself in terms of legitimacy and the moral positioning of status as a mental health service user. For example, in encounters with psychiatrists there appeared to often be no option but to disclose or to respond to directive suggestions.

*I kind of feel like they pat me on the head and say, yes, yes, yes, good little boy, but no, you’re staying on the CTO.***ID20, male, 1**^**st**^**time point**

The avoidance of relational work with ‘unnecessary’ or excessive numbers of human network members (see earlier themes) might account in part for the salience placed on inanimate objects, places, activities. In the quote below, the connections most foregrounded as supporting mental health management by ID8 were objects such as the radio, newspapers and magazines.

***It’s about whoever or whatever you feel is important to you, day to day, kind of keeping on top of managing [your mental health] …***

*Day to day. Well the radio. I live on my own so I play the radio quite a lot.*

***Yes? And would that go in the middle [circle]?***

*Yes, the radio. Yes.*

***Anything else? Or anybody else?***

*… next one will be newspapers, newspapers magazines. And also … on the third one I would say shopping, yes. Yes, yes, local shops and shopping. And, who else will there be? I play music. I play CDs and cassettes.***ID8, male, 2**^**nd**^**time point**

Engaging with objects, music and hobbies on an individual basis was considered an important source of distraction, intellectual stimulation and reliability of connection through being able to secure these with minimal amounts of navigation. As such, the value of these types of network members remained stable over the 12 months follow-up period.

*The hobbies are a good distraction really, more than anything. I suppose the hobby is kind of a distraction technique to when I want to self-harm, so it either postpones that self-harming, which then reduces the intensity, because the feelings have died down, do you see what I mean.***ID28, female, 1st time point**

These types of network ties also at times had utility as transactional objects in creating, mediating and strengthening relationships with others. Engaging with other people through hobbies and valued activities was an important source of ‘low intensity’ social interaction or connection to weaker ties which was considered beneficial to mental health and an important source of normalisation.

*Does your art group help?*

*It’s a nice group of largely older ladies, but not all older ladies, there’s some chaps there, there’s some younger people and, err, it’s my, sort of, social morning, because I … I’ve been going now for quite a long time and, err, I know people there and I feel safe in it, it’s not … I’m not really good in groups normally, but, err, it’s a group I feel safe with.*

*And why is that social side important to you in terms of your mental health would you say?*

*It helps you feel normal.***ID24, female, 1**^**st**^**time point**

*The gym would be my second most important circle. Again, for the reason that I felt if I exercised it was a way of meeting people outside of work, and just to make myself feel good in terms of my body image, because I do think I'm carrying more weight than I've carried ever in my life.***ID15, female, 1**^**st**^**time point**

## Discussion

We conducted a qualitative study of personal support networks informed by the Network Episode Model to gain an understanding of the nature and negotiation of supportive relationships undertaken by people diagnosed with a severe and enduring mental health condition. Our longitudinal qualitative data highlights the nature of the relational work required to negotiate support from network members and the value attributed to different types of network member. The reasons underpinning and sustaining such differences are fundamentally important to consider. The results provide new understanding of the experience of leveraging support from personal support networks and the posited ‘limitations’ in social networks of people with severe and enduring mental health problems [19, 20]. The findings provide new learning to help service providers address the prevailing dissatisfaction with formal mental health service provision [42–44].

Contemporary mental health service provision fails to consider important social relations and connections which are important for providing support to those with severe and enduring mental health problems [1]. This study lends further support to this assertion by identifying the potential value attributed to health professionals by participants but the limited actual contribution in the everyday management of mental health. The combination of qualitative methodology with a personal support network approach informed by the Network Episode Model provided an opportunity to further understand the central elements of support and management that are accessed but remain largely invisible for those delivering care in formal services. Recently developed interventions designed to understand and enhance social networks in addition to promoting community engagement such as Community Navigators [45], Connecting People [46] and GENIE [47] are currently being trialled and may go some way towards health services addressing these identified needs.

Participants in the current study described relatively small personal support networks and the often-precarious nature of social ties. This supports existing evidence that those with mental health problems have smaller networks of reduced quality [19, 20]. The study adds to existing evidence by identifying potential reasons for this which included decisions made by participants not to engage in the significant work required to sustain such relationships and fractious current and previous relationships with other people often attributed to misunderstanding and miscommunication in relation to their mental health condition. The intertwining of practical and moral dilemmas in identifying, offering, accepting and rejecting support is salient and explicit in the accounts of personal network support for participants in the current study which is likely to impact on the propensity to seek help from people in their network. The relational work required to engage others appeared hard, contingent and vicarious because of felt and enacted stigma [26, 27]. Developing relationships with others was hindered by a lack of confidence fuelled by the experience of mental illness and the fear of rejection or failure.

The study provides further support for the Network Episode Model through its demonstration of the dynamic and social nature of mental illness management [9, 12]. Participants described nuanced strategies which were employed in the face of the aforementioned dilemmas in order to manage mental illness effectively. This supports previous studies using the NEM which have found that people actively seek out the most effective discussants to talk to about their mental health from a wide range of potential sources [18]. Novel strategies identified in the current study included the active restriction of personal support networks and of non-kin ties in particular especially during periods of acute illness. These findings echo more recent evidence which suggests that network constriction may in fact be an adaptive response which promotes recovery [29, 30]. Participants also identified strategies which reflected the flexibility of friendships including diversification or ‘thinning’ of friendships (e.g. focusing on one or two key network members to enable them to garner optimal social support and reduce burden). Participants in the current study often foregrounded distal friendships, valued activities, hobbies, places and things in their accounts of the supportive features of personal communities of support [32]. These findings extend the NEM by highlighting the strength of weaker ties and non-human network members for managing mental health problems. The value placed on objects and things in relation to mental health management aligns with capabilities approaches which aim ‘to achieve outcomes that people value and have reason to value” [48]. In this way, personal support networks provide the opportunity and freedom for people to access resources that are of value to them. Such preferences and choices show the hidden value of resources that are often backgrounded in traditional service encounters and indicate the need for reorientation of priorities when discussing care and self-management with service users [32, 49].

### Strengths and limitations

The study gains its strengths from the in-depth, longitudinal interviews with 29 service users which helped to elucidate the nature and negotiation of support from personal support networks. The personal communities approach enabled participants to self-select the network members that were important to them and they were not restricted in terms of numbers or types of network members. On average, interviews lasted approximately one hour which enabled participants to provide detailed descriptions of the value attributed to personal communities and how support was leveraged. The study may have benefitted from ethnographic observation of interactions with network members to further explore relationships between participants and their personal communities. Only the views of service users are presented here and it was therefore not possible to compare and contrast findings with data from identified network members. Participants were recruited from 7 mental health trusts in the UK, included only those cared for within the community and the vast majority were White British. The views reported here may therefore not reflect those of other ethnic groups or service populations. Data was not available on time since diagnosis or length of treatment which may have impacted on identified personal communities and the experience of mental health problems.

## Conclusion

Access to valued activities, hobbies and things should be considered alongside human network members in providing a means of ongoing support and resource for the management of severe mental illness. The former provides greater security without the need to negotiate and manage the stress and unpredictability of interaction and relationality with humans.

## Data Availability

The dataset(s) generated during the current study are not publicly available due to ethical restrictions but are available from the corresponding author on reasonable request.

## Appendix

### Examples of questions from baseline interview schedule

Questions asked of each identified network member:

- How does [network member] help you manage your condition day to day?
- What does [network member] do to help you cope with your illness?
- General questions asked in conjunction with network diagram:
- Where or to whom do you go to find out more about your illness?
- Is there anything else that you find useful to help you cope with your illness?
- When you need advice about, or help with, your diet, who do you go to?
- When you need advice about, or help with, exercise, who do you go to?
- Where would you go, or who would you go to, for advice or help with relieving stress?
- When you need advice about, or help with, medications, who would you turn to?
- Living with a long-term condition often means that you need to do things more slowly, take on additional tasks and other people may need to make compromises that are good for your health. Who in your diagram does these things?
- Who do you turn to when you are worried about your illness?
- Looking at your diagram, who do you think you would like to be more involved in helping you with your illness than they are at present?
- What and who helps or hinders your care? Can you think of examples?
- Who or what (e.g. people/pets) in your diagram gives you emotional support and encouragement? Can you think of examples?
- Who in your diagram would step in/stand up for you when you do not feel well enough to stand up for yourself?
- Who among the network members in your diagram do you help? How?

## Abbreviations

CPA: Care Planning Approach
CPN: Community Psychiatric Nurse
UK: United Kingdom
